# What Is the Link Between Migraine and Hypothyroidism? A Systematic Literature Review

**DOI:** 10.3390/jcm14134645

**Published:** 2025-07-01

**Authors:** Martyna Michalik, Justyna Łapicka, Marcin Sota, Julia Zawieska, Olga Grodzka, Katarzyna Kępczyńska

**Affiliations:** 1 Department of Neurology, Faculty of Medicine and Dentistry, Medical University of Warsaw, Ceglowska 80, 01-809 Warsaw, Poland; s080145@student.wum.edu.pl (M.M.); katarzyna.kepczynska@wum.edu.pl (K.K.); 2 Doctoral School, Medical University of Warsaw, Ceglowska 80, 01-809 Warsaw, Poland

**Keywords:** autoimmune diseases, Hashimoto’s disease, hypothyroidism, migraine, primary headache disorders

## Abstract

**Background**: Hypothyroidism is defined as a deficiency of thyroid hormones and is further classified into primary, secondary, and tertiary types, based on the root cause of the deficiency. Migraine is a primary headache disorder, characterized by unilateral, pulsating pain, lasting from 4 to 72 h, accompanied by symptoms such as photophobia, phonophobia, nausea, and emesis and sometimes preceded by specific aura phenomena. Both diseases are more prevalent in women than in men. While the primary focus of this systematic review was on the relationship between hypothyroidism and migraine, we also included relevant data on headaches in general when they provided valuable context or mechanistic insight. **Methods**: This systematic review aimed to summarize the current knowledge about the relationship between migraine and hypothyroidism. The Preferred Reporting Items for Systematic Reviews (PRISMA) guidelines were applied. Screening of two databases led to including 29 relevant studies in the review. **Results**: Studies demonstrated that migraine and disturbed thyroid function may influence one another. The positive correlation between migraine and hypothyroidism, mainly Hashimoto’s disease, was presented in several studies. Moreover, some research identified this correlation in pediatric populations. Finally, the effects of levothyroxine use, a treatment applied in hypothyroidism, on migraine course were presented. **Conclusions**: A better understanding of the correlation between migraine and hypothyroidism may lead to an increase in the understanding of the pathogenesis of both disorders and positively impact clinical practice.

## 1. Introduction

### 1.1. Background About Migraine

Migraine is a common disabling primary headache disorder, in most cases episodic, that usually lasts 4–72 h. It is accompanied by nausea, vomiting, and/or photophobia and phonophobia [1]. This relatively frequent and common disease affects women more often than men, with a sex prevalence ratio of 3:1 [2]. The highest proportion of patients falls within the 30–40-year age group.

According to the Headache Classification Committee of the International Headache Society, migraines can be classified into the following three main subtypes: migraine with or without aura and chronic migraine (CM) [1,3,4]. In migraines with aura, the pain usually lasts 4–72 h, is unilateral and pulsatile with photophobia and phonophobia, and is associated with visual, sensory, motor, and speech disturbances [1,5]. In migraines without aura, the pain characteristics remain the same as in migraines with aura, but without preceding aura symptoms. CM is defined as pain for 15 or more days per month, with at least 8 days characterized by typical migraine headaches, which last for more than 3 months [1,6].

For the acute treatment of migraine attacks, oral non-steroidal anti-inflammatory drugs, triptans, and a novel class of drugs, namely gepants and diants, are recommended. For migraine prophylaxis, traditional medications such as beta-blockers, flunarizine, valproic acid, and topiramate, along with new treatments, including monoclonal antibodies against calcitonin gene-related peptide (CGRP), gepants, and botulinum toxin, are considered first-choice options [7,8].

### 1.2. What Is Hypothyroidism?

Thyroid gland disorders are among the most common endocrine diseases in the world. Hypothyroidism is defined as a deficiency of thyroid hormones and can be further classified as primary, when the dysfunction of the thyroid gland itself causes decreased production of thyroid hormones; secondary, due to inadequate thyroid-stimulating hormone (TSH) production by the pituitary gland; and tertiary, as a consequence of diminished thyrotropin-releasing hormone (TRH) production by the hypothalamus [9]. The most common cause of hypothyroidism is chronic autoimmune thyroiditis, associated with iodine deficiency, which, to the greatest degree, occurs in older women [10,11]. Additionally, drug therapies, especially amiodarone, lithium carbonate, radioactive iodine treatment, and invasive procedures such as thyroid surgery, have been proven to lead to hypothyroidism [12,13,14].

This disease occurs in 5% of the human population, accounting for both the overt and subclinical types, with ten-fold higher rates in women compared to men, making it a significantly more prevalent condition in females [15]. It affects almost every single organ of the body and has a wide variety of clinical presentations [16,17,18]. Most symptoms are nonspecific. Therefore, the diagnosis is based on serum thyroid function tests via biochemical laboratory diagnostics [19,20]. Additionally, the thyroid dysfunction can be subclinical, with the thyroid hormone levels in the normal range and no obvious clinical manifestation. Some studies suggest that even a subclinical dysfunction may be related to diverse disorders [21]. Thus, subclinical thyroid dysfunction should not be omitted from any analyses. The current standard of treatment for patients suffering from hypothyroidism is levothyroxine monotherapy, which is highly effective, safe, and inexpensive [22]. However, 10% of patients undergoing pharmacological therapy display symptoms of the disease despite normalization of biochemical markers [9].

Bilateral and non-pulsatile headaches occur to a greater degree in people suffering from hypothyroidism and remain after the correction of thyroid hormone levels by pharmacological treatment [23,24]. The current understanding of the mechanism behind these headaches is not clear. Additionally, a recent study showed a link between hypothyroidism and more severe unilateral, pulsatile headaches with nausea or vomiting [1,25], which can usually be considered migraine.

### 1.3. The Purpose of the Review

Therefore, this review aimed to summarize the current knowledge regarding the link between migraine and hypothyroidism in a systematic manner. Given the growing interest in the possible relationship between neurological and endocrine disorders, a comprehensive review of existing evidence seems to be necessary. By providing an organized analysis of available studies, we hope to clarify current understanding, highlight research gaps, and support future investigations in this important field. This review is centered on the link between hypothyroidism and migraine; however, we also incorporated findings related to general headache disorders when they enriched the clinical context or elucidated underlying biological pathways.

## 2. Methodology

This systematic review was written in accordance with the Preferred Research Items for Systematic Reviews and Meta-Analyses (PRISMA 2020). The first step was registration in Prospero, the International Prospective Register of Systematic Reviews, with the registration ID: CRD42024607171.

### 2.1. Inclusion and Exclusion Criteria

To select the most suitable articles, we applied criteria for inclusion and exclusion. Study designs such as randomized controlled trials and observational studies (cohort, case-control, and cross-sectional) were included. We considered only research on the predetermined topic. Moreover, articles had to be written in English to be analyzed.

On the contrary, the following study designs were removed: reviews and meta-analyses, case reports, case series, commentaries, editorials, and conference abstracts. We excluded meta-analyses to avoid potential duplication of primary data, as most of the included original studies may already be represented in such reviews. This decision was made to preserve the clarity and integrity of the dataset by focusing exclusively on primary research and ensuring consistency in data extraction and analysis. Finally, studies that did not cover the review topic were naturally excluded.

### 2.2. Selection Process

Two authors (MM and JŁ) performed the selection independently, which was followed by an assessment of discrepancies by another author (OG) to avoid bias.

**Figure 1:**
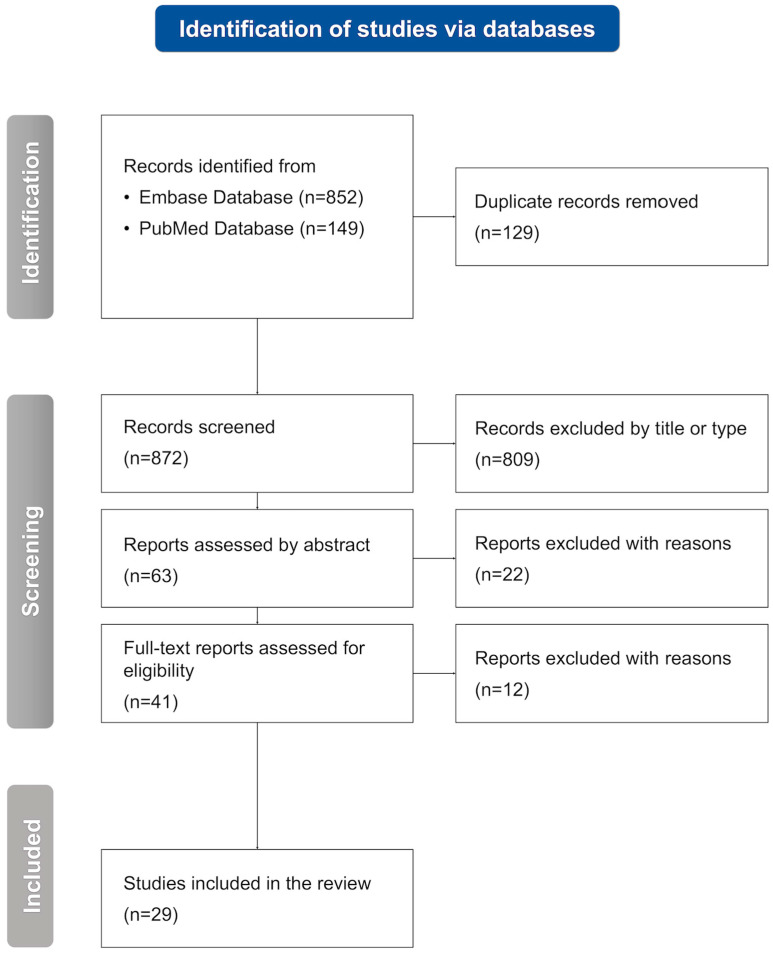
A flowchart of the selection process leading to the inclusion of 29 original studies in the systematic review; n, number of studies.

The initial search resulted in indicating 1001 records from two databases, PubMed (149) and Embase (852), using the following search strategy: (migraine) AND (hypothyroidism OR Hashimoto). After duplicate removal, 872 studies were left for consideration. Screening by title or type resulted in the exclusion of 809 inappropriate studies. In the further abstract analysis, 22 reports were removed. Finally, the full-text assessment for eligibility led to the exclusion of another 12 articles. Eventually, 29 studies were included in the systematic review. The selection process is presented in the flowchart (Figure 1). A thorough analysis of the chosen articles is provided in Section 3 below.

### 2.3. Data Extraction and Data Synthesis

To ensure a comprehensive understanding of the available evidence, relevant data from the included studies were carefully reviewed and extracted. The process was conducted in a structured manner, aiming to capture all information necessary for addressing the review objectives. Attention was given to maintaining consistency and accuracy, with an emphasis on key aspects of the review topic. Due to heterogeneity in study designs, populations, and outcomes, a narrative synthesis was conducted. The findings were grouped according to the theme and summarized to highlight similarities and differences across studies. This approach allowed for a comprehensive understanding of the evidence, despite some variations in methodology and design.

## 3. Findings

### 3.1. Correlation Between Thyroid Function and Headaches in General

The main focus of the systematic review is the correlation between migraine and hypothyroidism. However, we distinguished the following section, since some valuable studies analyzed how thyroid function and headaches are linked in general. Thus, exploring this issue in more depth provides the opportunity to better understand the correlation between migraine and hypothyroidism, which is explored in the paragraph below. The link between thyroid function and headache has been well-documented, though it still remains complex. New research posits that hypothyroidism is significantly associated with headache disorders, including migraine. Some studies have suggested a bidirectional relationship. Several studies have shown that migraine headaches and hypothyroidism can co-occur more frequently than they occur individually in the general population.

Lima-Carvalho et al. [26] conducted a study in which they analyzed the frequency of headaches attributed to hypothyroidism among a group of hypothyroid patients. The researchers evaluated the differences in clinical presentation compared to the group without headache and assessed outcomes after the hypothyroidism had been controlled with levothyroxine. They found that headaches are associated with hypothyroidism and that approximately one-third of hypothyroid patients reported headaches. According to headache characteristics, 63% of cases were pulsatile, 78% of cases lasted from 4 to 72 h, 47% had a unilateral location roughly half of the time, and 60% of patients experienced nausea or vomiting. In 92% of patients, these headaches were more consistent with migraines than with the typical presentation of hypothyroidism-associated headaches (HAHs) as defined by ICHD-3 criteria. Finally, the study showed that levothyroxine treatment could relieve headaches in 78% of patients and thus supported the conclusion that hypothyroidism may cause headaches, including migraine. Martin et al. [27] explored the relation between thyroid function and headaches. In their study, 824 individuals without prior thyroid disease were followed for an average of 12.6 years, with an outcome that led to a significant association between headache disorders, including migraine, and the development of new-onset hypothyroidism. For people with headaches, 8.2% developed hypothyroidism, compared to only 6.2% for those without headaches (HR 1.21, 95% CI: 1.001, 1.462). According to the researchers, this indicates an additional risk of 21% for headache development. For those who possibly had migraine, an even higher risk of hypothyroidism was observed (HR 1.41, 95% CI: 1.009, 1.973), with an incidence of 10.8%. The researchers conclude that there is a possibility of a two-way interaction between headaches and thyroid dysfunction, mediated through immune, autonomic, or genetic pathways.

Interesting findings, which stand in contrast with previous studies, were described by Harbeck et al. [28]. According to the researchers, TSH deficiency was associated with a significantly decreased incidence of headaches. More precisely, 25% of TSH-deficient patients reported headaches compared to 42.5% in normal TSH. Additionally, adrenocorticotropic hormone (ACTH)-deficient patients had a significantly lower headache rate (24.5%) compared to those with a proper level of ACTH secretion (43.5%). A similar pattern was observed in growth hormone (GH)-deficient versus GH-normal patients (22.5% versus 42.0%). In contrast, the prevalence of headaches was not significantly different between the hypothalamic–pituitary disorders group and the cardiovascular disease control group, indicating that factors other than hormone dysfunction play a significant role in headache pathogenesis in these patients. Additionally, hormonal excess syndromes, including those affecting the thyroid, were associated with a higher incidence of headache. These findings suggest that certain hormone deficiencies may, in fact, reduce the occurrence of headaches. All studies mentioned in this paragraph have been summarized, along with additional information, in Table 1.

### 3.2. Migraine and Hypothyroidism Prevalence

Furthermore, many recent studies indicate that migraines, specifically, and hypothyroidism are more commonly found together than separately in the general population. Abou-Elmaaty et al. [29] conducted research aimed at investigating this correlation. The study showed that 30.8% of patients with migraine had either subclinical or overt hypothyroidism, a significantly higher percentage in comparison to the control subjects. It was also shown that patients with migraine and tension-type headache (TTH) were 3.73 times more likely to develop hypothyroidism. Moreover, in 10.6% of the patients with migraine examined in the study, thyroid morphology on ultrasound was more abnormal compared to healthy study participants. Further supporting this, Khan et al. conducted research on migraine and hypothyroidism prevalence in patients in India [30]. Participants with primary headache disorders were tested for serum TSH and serum thyroxine. Hypothyroidism was found in 29 out of 86 patients with migraine, which was significantly more frequent than in the control group without migraine [2]. A similar study conducted by Matias-Guiu et al. [31] investigated factors associated with the differences in migraine prevalence in Spain. Conversely, hypo/hyperthyroidism was found to be negatively correlated with headache prevalence; however, the results might be biased by age. An additional study providing evidence of a correlation between migraine and hypothyroidism was conducted by Wouters et al. [32]. Their study demonstrated that thyroid hormone users had more comorbidities than nonusers. Individuals using thyroid hormone exhibited higher morbidity, particularly in terms of migraine. The headache appeared in 23.7% and 17.8% of thyroid hormone users and nonusers, respectively.

Muddasir et al. [33] investigated the comorbidities and health-related quality of life in patients with hypothyroidism. In this research, a structured questionnaire and evidence-based approach showed that among the physical comorbidities, hypothyroidism co-occurred in 9.9% of patients with migraine. Other researchers, including Panconesi et al. [34], also evaluated the prevalence of other diseases in patients with migraine based on detailed semi-structured interviews and health information from the database of specialist visits and hospitalization or the Emergency Department. In this study, hypothyroidism co-occurred in 6.4% of migraine patients. In addition, Tietjen [35] conducted a different study on migraine comorbidity constellations. He identified three groups of patients with migraine based on collected information on comorbid diagnoses and some environmental factors. In one of the groups, hypothyroidism was present alongside hypertension, hyperlipidemia, and diabetes mellitus. Hypothyroidism occurred in 27% of the patients included in this group. Interestingly, this particular group had more males, were older, and had a later age of headache onset than the other two migraine groups.

A slightly different study was conducted by Tasnim et al. [36], who investigated the relationship between migraine and thyroid function in the context of genetic background. Data from genome-wide association studies provided evidence of this genetic correlation. Particular genes at certain loci were identified as significant in their gene-based analysis. These studies, although with slightly different designs, lead to the conclusion that hypothyroidism is one of the most common comorbidities associated with migraine. All studies mentioned in this paragraph have been summarized, along with additional information, in Table 2.

### 3.3. Comparison Between Migraine and Other Headache Types

Migraine patients can be compared not only to healthy individuals, but also to other headache groups. Many researchers have compared migraine with various types of headaches in their works in terms of their correlation with hypothyroidism. In a study conducted in Bangladesh by Bhattacharjee et al. [39], researchers investigated the association between low thyroid hormone levels and migraine in adults. Subclinical hypothyroidism was more common in migraine patients than in non-migraine headache patients. Moreover, hypothyroidism was more closely associated with migraine than with other headaches, such as TTH. TSH levels were significantly higher in people with migraines than in those with other types of headaches. Another study conducted in Mexico by two researchers, L. E. Fernández-Garza and A. Marfil [40], analyzed an association between hypothyroidism and different headache types. Hypothyroidism was more common in patients with occipital (6.5%) and trigeminal (6.1%) neuralgia than in those with TTH (3.9%) and migraine (3.2%). However, patients with CM, specifically, had the highest prevalence of hypothyroidism (10.7%). Therefore, the researchers suggested that hypothyroidism may be a risk factor for chronic headaches, particularly in patients with cranial neuralgias.

In the research conducted by Gozubatik Celik et al. [41] in Turkey, various types of headaches were found in patients with Hashimoto’s disease (HD), particularly in those with subclinical HD. Headache was reported by 61% of patients with HD, with migraine, new daily persistent headaches (NDPH), and TTH being the most common. The researchers found that the headache associated with hypothyroidism was usually unilateral, most often fronto-orbital or temporal, pulsatile, and moderate to extreme in severity. Finally, the study conducted in the United States by Bigal et al. [42] identified somatic and lifestyle factors associated with the development of CM and NDPH. As in the previous analysis, it was suggested that hypothyroidism may be associated with CM and NDPH. In addition, these diseases are associated with factors such as allergies, asthma, caffeine, and alcohol consumption. These studies collectively suggest a strong association between hypothyroidism and various types of headaches, especially migraines and cranial neuralgias, with subclinical hypothyroidism being more prevalent among migraine patients. Additionally, hypothyroidism may contribute to the chronification of headaches, particularly in conditions such as CM and NDPH. All studies mentioned in this paragraph have been summarized, along with additional information, in Table 3.

### 3.4. Comparison Among Migraine Types

Further analyses of migraine should naturally focus on different migraine types. This division into episodic migraine (EM) and CM depends on the total number of headache days per month. EM should last 14 days or fewer, while CM occurs on 15 days or more, of which eight have to meet the criteria of typical migraine pain [1]. As already mentioned before, it has been suggested that hypothyroidism can be associated with migraine chronification. In this paragraph, we focus on this possible phenomenon. In their research, Filipchuk et al. [43] analyzed the medical records of EM and CM patients treated for hypothyroidism. The study showed that treated hypothyroidism was present in 29.55% of patients with CM, which was significantly higher compared to 8.96% in patients with EM. Similar results were reached by Nowaczewska et al. [44] in their study on the relationship between migraine and Hashimoto thyroiditis. Migraine patients with HD experienced longer durations of migraine, more monthly migraine days, and were more likely to develop CM. This leads to the conclusion that thyroiditis may influence the course of migraine, contributing to its chronification. Another researcher, Bigal et al. [42], intended to identify factors associated with the onset and transformation to chronic daily headaches. He compared somatic and lifestyle factors in the different types of headaches. Strong correlations were identified between patients with CM and hypothyroidism.

In contrast to the previous researchers, Spierings et al. [45] observed hypothyroidism to be equally common in both groups: 11.1% in EM and 11.8% in CM. This does not support the findings regarding the role of hypothyroidism in migraine chronification. What is more, considering the menstrual-cycle disorders associated with migraine, the research showed that hypothyroidism is related to menorrhagia. However, CM is possibly more often seen in dysmenorrhea. Furthermore, comparing these results with the study by Togha et al. [46] on characteristics and comorbidities of headache in patients over 50, it can be suggested that EM and CM are not dependent on hypothyroidism. According to the researchers, hypothyroidism affected 25.3% of patients with EM and 24.6% of those with CM. However, a significant rise in the prevalence of this thyroid condition was observed among participants with medication-overuse headaches (44.4%). The association between hypothyroidism and primary headache subtypes was examined by Spanou et al. [47] in a slightly different study. The researchers showed that the prevalence of any type of thyroid disorder in participants was overall 20.8%, and 6.3% of patients reported hypothyroidism. However, the analysis did not reveal any significant links between the headache subtypes and thyroid dysfunction.

On the other hand, the difference between the occurrence of hypothyroidism in migraine with aura and migraine without aura was noted by Li et al. [48]. The researchers conducted the study on the causality between multiple autoimmune disorders and migraine and its subtypes. They found a positive association between hypothyroidism and migraine without aura. A similar study was conducted by Rubino et al. [24]. In contrast to the former researchers, the latter noticed a link between hypothyroidism and both types of migraine, with and without aura. Nonetheless, migraine patients without aura presented hypothyroidism more frequently (in 37.75%) in comparison with migraine patients with aura (in 8.61%), which does not contradict the previous study. All studies mentioned in this paragraph have been summarized, along with additional information, in Table 4.

### 3.5. Levothyroxine Intake and Migraine Course

Furthermore, beyond migraine therapy, the topic of both pharmacological and non-pharmacological treatment has received increased attention recently. Levothyroxine replacement therapy, the standard treatment for hypothyroidism, has been reported to improve migraine severity in several studies. Dev et al. investigated the efficacy of a low fixed-dose thyroid replacement therapy as an additive treatment for migraine patients with subclinical hypothyroidism [49]. The study revealed a reduction in assessed parameters, including headache frequency, severity, duration, Migraine Disability Assessment Score (MIDAS), and MIDAS grade, at the three-month follow-up in the treatment group compared to the placebo group. Moreover, a reduction in headache duration was also observed in the levothyroxine group, though the difference was not statistically significant. Additionally, according to a study performed by Hepp et al., patients with hypothyroidism who were nonadherent to levothyroxine therapy were more likely to suffer from migraines compared to adherent patients [50]. Mirouliaei et al. evaluated the effect of levothyroxine therapy on migraine management in pediatric patients with subclinical hypothyroidism [51]. A total of 25 children with migraine headaches were monitored over a two-month treatment period. This study demonstrated that treating subclinical hypothyroidism effectively reduced both the severity and monthly frequency of migraine headaches. Additionally, in the already mentioned study by Lima-Carvalho et al. [26], levothyroxine treatment relieved headaches in 78% of patients. These studies provide insight into the complex relationship between thyroid function and migraines. Proper thyroid hormone regulation may have a therapeutic role in migraine management by reducing its severity, frequency, and duration. All studies mentioned for the first time in this paragraph have been summarized, along with additional information, in Table 5.

### 3.6. Hypothyroidism and Migraine in the Pediatric Population

Finally, what is usually considered separately is the course of various diseases in children. Several studies investigated the association between hypothyroidism and migraine in the pediatric population, with one already described [51]. The study conducted by Fallah et al. analyzed the occurrence of subclinical hypothyroidism among children aged 5 to 15 years suffering from migraine headaches [52]. It showed that 24% of the examined group (104 pediatric migraineurs) had subclinical hypothyroidism, which supported the comorbidity of these conditions [52]. Moreover, it was observed that children with migraines and hypothyroidism had a significantly higher frequency of headaches per month and longer headache duration. However, there was no significant difference between gender, mean age, age of migraine onset, or headache severity between the two groups. Similarly, the research of Hassan et al. evaluated the correlation between subclinical hypothyroidism and childhood migraine [53]. In their study, a total of 200 children and adolescents were evenly split into two groups, and their clinical and biochemical parameters were analyzed statistically. The results demonstrated that migraineurs exhibited higher TSH but normal thyroxine, with subclinical hypothyroidism being more prevalent compared to controls (17% vs. 2%). In contrast, the study showed that thyroid function was not correlated with the occurrence of aura. In addition, the research assessed body mass index (BMI), showing that obesity and being overweight were more frequent among children with migraine (8% and 5% vs. 2% and 1%, respectively). Among the migraine patients, 77% of overweight and obese individuals had subclinical hypothyroidism, and 15.4% had overt hypothyroidism, compared to 8% and 0%, respectively, in those with normal body weight. This suggests that assessing thyroid function in children with migraines, particularly those with a high BMI, may be a reasonable approach in diagnostic evaluations. Additionally, the researchers evaluated the prevalence of nodular goiter in both migraine and control groups, finding no significant difference.

Consistently, Kang et al. discussed the clinical manifestations of headaches in children under 7 years of age [54]. From the total number of 146 children included, only one patient had subclinical hypothyroidism. However, despite ongoing debate and the presence of only one patient with an abnormal TSH level in the study, the researchers suggest that a potential link between headache and TSH levels remains, and according to them, future research should explore this association with a larger sample of high-TSH subjects. Moreover, the study also indicates that preschool-aged children typically experience headaches with a benign course, and secondary headache etiologies can be determined through different diagnostic approaches.

In contrast to all the studies presented, Ekici et al. argued against a correlation between migraine and subclinical hypothyroidism [55]. In the study, only five children (5.1%) were found to have subclinical hypothyroidism from a total of 98 participants with migraine, all of whom were in the prepubertal stage.

The relationship between hypothyroidism and migraine in the pediatric population remains a subject of ongoing investigation. While several studies have reported a higher prevalence of subclinical hypothyroidism among children with migraines, other research has found no significant correlation. The findings suggest that migraineurs, particularly those with obesity or overweight status, may be more likely to exhibit thyroid dysfunction, emphasizing the potential importance of thyroid function assessment in specific cases. However, inconsistencies in the available data highlight the need for further large-scale studies to clarify the extent of this association and its clinical implications. All studies mentioned in this paragraph have been summarized, along with additional information, in Table 6.

## 4. Discussion

Hypothyroidism and headache, particularly migraine, share a complex relationship, with new evidence pointing towards a bidirectional relationship. Clinical observations indicate that approximately one-third of individuals with recent-onset hypothyroidism experience migraine-like headaches that improve in most cases after levothyroxine treatment. Additionally, longitudinal data suggest that patients with preexisting headache disorders have an increased risk of developing subsequent hypothyroidism. Studies have shown that migraines and hypothyroidism frequently co-occur, as confirmed by numerous analyses in which the percentage of migraine patients with thyroid disorders was significantly higher than that in the control group. Some studies suggest that migraines increase the risk of developing hypothyroidism, while others indicate potential genetic factors underlying this correlation. Despite some differences in findings, there is strong evidence that hypothyroidism is one of the most common comorbidities in individuals suffering from migraines. What is interesting is that, according to particular studies, patients with certain pituitary hormone deficiencies, e.g., TSH, ACTH, and GH, demonstrate a lower prevalence of headaches. Moreover, researchers suggest a potential link between hypothyroidism and the progression of EM to CM, as a higher prevalence of thyroid dysfunction has been observed in CM patients. However, findings on this relationship remain inconsistent, with some studies indicating no significant association. Ideally, more studies on a larger patient sample should be conducted. Additionally, differences in hypothyroidism prevalence have been noted between migraine subtypes, particularly between migraines with and without aura. These results suggest that hypothyroidism is more often associated with migraine without aura; however, it is also observed in patients with migraine with aura.

These findings indicate a significant association between hypothyroidism, particularly in its subclinical form, and various types of headaches. This correlation was observed particularly between hypothyroidism and cranial neuralgias, such as occipital or trigeminal neuralgia, while secondary headaches were less commonly reported. Migraine patients have been found to exhibit higher rates of subclinical hypothyroidism and elevated TSH levels compared to individuals with other headache types, such as TTH. These reports suggest that hypothyroidism may contribute to both the development and chronification of certain headache disorders, highlighting the importance of thyroid function screening in patients with persistent or treatment-resistant headaches. However, not all research supports this thesis; thus, more studies should be performed on the influence of thyroid hormones on migraine chronification.

Another critical issue is the relationship between hypothyroidism and migraine in the pediatric population, which remains a subject of ongoing investigation. While several studies have reported a higher prevalence of subclinical hypothyroidism among children with migraines, other research has found no significant correlation. Findings suggest that migraineurs, particularly those with obesity or overweight status, may be more likely to exhibit thyroid dysfunction, emphasizing the potential importance of thyroid function assessment in specific cases. The inconsistent results observed in studies involving children may be influenced by several factors. One important consideration is hormonal variability related to age, particularly during puberty. Rapid endocrine changes could affect both thyroid function and migraine symptoms, making it harder to detect clear associations in cross-sectional analyses. BMI may also play a role, as some studies have reported a higher incidence of thyroid dysfunction in overweight or obese children with migraine. This suggests a potential link between metabolic status and thyroid regulation. Taken together, these factors could help explain the variability in findings and highlight the need for future large-scale studies to account for age and BMI when examining this relationship.

The observed association between hypothyroidism and migraine warrants careful interpretation. Although the results suggest a link between the two conditions, the cross-sectional and observational nature of the available data precludes definitive conclusions regarding causality. Several biological mechanisms may underlie this relationship, including dysregulation of serotonergic pathways, alterations in pain perception, or neuroinflammatory processes, all of which have been implicated both in thyroid dysfunction and in migraine pathophysiology [56,57,58]. Nonetheless, it remains unclear whether hypothyroidism contributes directly to the development or exacerbation of migraine, or whether the association reflects a coincidence arising from overlapping risk factors, such as female sex, hormonal fluctuations, or autoimmune predisposition. Given these uncertainties, the findings should be interpreted with caution. Future prospective studies and mechanistic research are needed to clarify whether the relationship is causal or merely associative.

## 5. Conclusions

The current evidence supports the existence of a significant association between hypothyroidism—including its subclinical form—and various primary headache disorders, most notably migraine, which was the main topic of this review. This relationship appears to be complex and potentially bidirectional, with overlapping pathophysiological mechanisms. The frequent co-occurrence of these conditions justifies increased clinical awareness. Screening for thyroid dysfunction in migraine or other headache patients, especially those with chronic, treatment-resistant, or atypical headache presentations, may provide additional information on diagnostic and therapeutic aspects.

**Figure 2:**
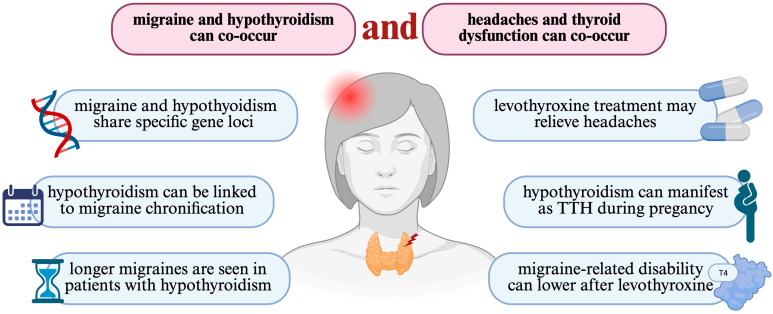
A graphical summary of some of the main aspects linking headaches and migraine with thyroid dysfunction. TTH, tension-type headache.

Finally, one of the most essential conclusions of this review is that optimal regulation of thyroid hormones through levothyroxine administration may have a therapeutic impact on migraine management by reducing its severity, frequency, and duration. Figure 2 summarizes the most important aspects mentioned in this review. These findings highlight the need for further longitudinal and mechanistic studies to better understand the interplay between hypothyroidism and migraine.

## 6. Limitations

This study has several limitations, which result from deliberate methodological choices made during the development of the protocol registered in PROSPERO. These decisions aimed to ensure consistency, clarity, and feasibility in the review process. Firstly, the search was conducted across two databases, which could lead to missing an important article not indexed in either database. However, both databases are well-recognized for their comprehensive coverage of biomedical literature. This decision ensured the inclusion of high-quality peer-reviewed studies while maintaining a reproducible and transparent search strategy. In addition, we did not include meta-analyses; however, we believe that all the important primary data were included in the original research, which we considered. Moreover, we included only studies written in English. Although including non-English studies could have broadened the dataset, language restrictions were necessary due to resource constraints and to avoid potential translation bias or misinterpretation. The applied search strategy was simple, and although multiple articles were found, there remains a risk of missing some. Finally, the number of studies is limited, and the number of patients in each study was sometimes relatively small. More research is needed. Despite these limitations, our systematic review adheres to established methodological standards and provides a reliable synthesis of current evidence on the relationship between hypothyroidism and migraine. We believe that our findings can serve as a valuable basis for future research, including more extensive reviews with broader inclusion criteria.

## Figures and Tables

**Table 1:** A summary of studies examining the overall correlation between thyroid function and headaches.

Ref.	Year	Population	Comparison	Findings	HT Diagnosis
Lima-Carvalho et al. [26]	2017	73 pts with HT of recent onset and HAH	140 pts with HT of recent onset without HAH	53% HAH pts had a prior history of MIG compared to 38% non-HAH pts; after 12 months of levothyroxine therapy, 78% of patients with HAH had a reduction in headache frequency or complete remission	assessment of thyroid hormones levels
Martin et al. [27]	2017	2021 pts with headache disorders	6391 pts without headache disorders	21% increased risk of developing new onset HT in the study group; 41% increased risk of developing HT in pts with possible MIG	assessment of thyroid hormones levels
Harbeck et al. [28]	2015	121 HPD pts	115 CD pts	ACTH deficiency pts had a headache prevalence of 24.5% compared to 43.5% in normal ACTH pts; pts with GH deficiency had a headache prevalence of 22.5% compared to 42% in normal GH pts; pts with TSH deficiency had a headache prevalence of 25% compared to 42.5% in normal TSH pts	assessment of thyroid hormones levels

ACTH, adrenocorticotropic hormone; CD, cardiovascular disorders; GH, growth hormone; HAH, headache attributed to hypothyroidism; HPD, hypothalamic–pituitary disorders; HT, hypothyroidism; MIG, migraine; pts, patients; Ref., Reference; TSH, thyroid-stimulating hormone.

**Table 2:** A summary of studies on the prevalence of migraine and hypothyroidism.

Ref.	Year	Population	Comparison	Findings	HT Diagnosis
Abou-Elmaaty et al. [29]	2020	94 MIG pts	100 HC	subclinical or overt HT in 30.8% MIG pts vs. 10% HC; abnormal thyroid ultrasound in 10.6% MIG pts vs. 3% HC	assessment of thyroid hormones and thyroid ultrasound
Khan et al. [30]	2015	86 MIG pts	500 HC	HT in 33.7% MIG pts vs. 11.2% HC with subclinical HT and 1.2% HC with HT	assessment of thyroid hormones
Matias-Guiu et al. [31]	2014	31,300 households		negative correlation between MIG pts and hypo/hyperthyroidism	database of the National Statistics Institute of Spain
Wouters et al. [32]	2023	4537 thyroid hormone users among 147,201 adult pts	142,664 nonusers	MIG in 23.7% thyroid hormone users vs. 17.8% nonusers	questionnaires, physical examination, biochemical measurements, and verified medication use
Muddasir et al. [33]	2020	323 MIG pts	-	9.9% MIG pts with HT	questionnaire
Panconesi et al. [34]	2015	1000 MIG pts	-	6.4% MIG pts with HT	survey
Tietjen et al. [35]	2007	223 MIG pts; 55 pts in group 1 (hypertension, hyperlipidemia, DM, and HT)		HT in 27% pts in group 1; group 1 had a higher proportion of males (22%), had an older median age (52 years) and had a later age of headache onset (median years: 22)	questionnaire
Tasnim et al. [36]	2023			a positive genetic correlation between MIG and HT, MIG and secondary HT, and MIG and fT4 levels	the GWAS summary statistics from the latest migraine GWAS [37]; statistics for HT, and secondary HT from PANUK Biobank; statistics for TSH and fT4 from GWAS data [38]

DM, diabetes mellitus; HC, healthy controls; HT, hypothyroidism; MIG, migraine; Ref., reference; vs., versus.

**Table 3:** A summary of studies on thyroid disorders in patients with migraine compared to those with other headache disorders.

Ref.	Year	Population	Comparison	Findings	HT Diagnosis
Bhattacharjee et al. [39]	2021	50 MIG pts	50 non-MIG pts	high TSH level higher in MIG pts compared to HC; subclinical HT more prevalent in MIG pts than in HC	survey, family history
L.E. Fernández-Garza et al. [40]	2020	792 pts with 1 headache diagnosis	73 pts with 2 headache diagnoses; 4 pts with 3 diagnoses	35 pts with confirmed HT diagnosis	clinical interview
Gozubatik Celik et al. [41]	2022	95 HD pts with headaches (38 HRH pts, 57 PH pts) (21 MIG pts, 17 TTH pts, 20 NDPH pts)	60 HD pts without headache disorders	subclinical HT in 22% with PH; overt HT in 7.2% with PH	clinical observation and interview
Bigal et al. [42]	2002	791 pts	399 CM pts had analgesic overuse, 158 CM pts without analgesic overuse, 69 NDPH pts, 100 EM pts, 65 CPTH pts	the transformation of episodic headaches into CDH, or the development of NDPH, was associated with factors beyond just medication overuse	HT diagnosed in people with CM, NDPH

CDH, chronic daily headache; CM, chronic migraine; CPTH, chronic posttraumatic headache; EM, episodic migraine; HD, Hashimoto’s disease; HRH, hypothyroid related headache; HT, hypothyroidism; NDPH, new daily persistent headache; MIG, migraine; PH, primary headache; pts, patients; Ref., reference; TTH, tension-type headache.

**Table 4:** A summary of studies on hypothyroidism in relation to different migraine types.

Ref.	Year	Population	Comparison	Findings	HT Diagnosis
Filipchuk et al. [43]	2022	67 EM pts	44 CM pts	HT in 8.96% EM pts vs. 29.55% CM pts	the medical records of MIG patients
Nowaczewska et al. [44]	2024	592 EM pts	336 CM pts;	CM in 47.2% pts with HD vs. 34.8% pts without HD; HD in 9.46% EM pts vs. 14.88% CM pts	the medical records of MIG patients
Bigal et al. [42]	2002	100 EM pts, 399 CM with MOH, 158 CM without MOH	69 de novo development of NDPH, 65 chronic posttraumatic headache	strong correlations between EM and CM pts and HT (odds ratios of 8.4)	the medical records of MIG patients
Spierings et al. [45]	2015	45 EM pts	51 CM pts	HT in 11.1% EM pts vs. 11.8% CM pts	questionnaire
Togha et al. [46]	2022	198 EM pts	61 CM pts	HT in 25.3% EM pts vs. 24.6% CM pts	questionnaire
Spanou et al. [47]	2020	253 MO pts, 49 MA pts	53 TTH pts, 29 MOH pts, 23 mixed-type headache pts (MA/MO and TTH), 9 CH pts, and 11 pts with other primary headaches	no significant association between headache subtypes and thyroid dysfunction; HT in 4% MIG pts	the medical records of MIG patients
Li et al. [48]	2024	3541 MA pts	3215 MO pts	a positive causal association between HT and MO	statistics from Europe’s largest genome-wide association study
Rubino et al. [24]	2018	151 HT pts	-	MO in 37.75% and MA in 8.61% HT pts	interview and biochemical parameters

CH, cluster headache; CM, chronic migraine; EM, episodic migraine; HD, Hashimoto’s disease; HT, hypothyroidism; MA, migraine with aura; MIG, migraine; MO, migraine without aura; NDPH, new daily persistent headache; Ref., reference; TTH, tension-type headache; vs., versus.

**Table 5:** A summary of studies on the influence of levothyroxine intake on migraine course.

Ref.	Year	Population	Comparison	Findings	HT Diagnosis
Dev et al. [49]	2023	43 MIG and subclinical HT pts treated with LT4	44 MIG and subclinical HT pts treated with placebo	↓ headache frequency and severity, ↓ MIDAS score, ↓ MIDAS grade in LT4 pts compared to placebo pts	assessment of thyroid hormones
Hepp et al. [50]	2018	159,314 HT pts nonadherent to LT4 treatment	159,314 HT pts adherent to LT4 treatment	study group more likely to suffer from MIG compared to control group	not given
Mirouliaei et al. [51]	2012	25 MIG and subclinical HT pts	before and after LT4 treatment	↓ headache frequency and severity after LT4 treatment compared to before	assessment of thyroid hormones

HT, hypothyroidism; LT4, levothyroxine; MIDAS, Migraine Disability Assessment Score; MIG, migraine; Ref., reference.

**Table 6:** A summary of studies on migraine and hypothyroidism in the pediatric population.

Ref.	Year	Population	Comparison	Findings	HT Diagnosis
Ekici et al. [55]	2015	98 pediatric MIG pts	-	only 5 pts with subclinical HT	assessment of thyroid hormones
Fallah et al. [52]	2012	104 pediatric MIG pts	-	25 pts with subclinical HT; ↑ headache frequency and duration in subclinical HT pts vs. those without	assessment of thyroid hormones
Hassan et al. [53]	2022	100 pediatric MIG pts	100 non-MIG pts	subclinical HT in 17 MIG pts vs. 2 non-MIG pts; overt HT in 2 MIG pts vs. 0 non-MIG pts; obesity and overweight more frequent in MIG pts vs. non-MIG pts subclinical HT in 77% obese/overweight MIG pts vs. 8% normal body weight pts; overt HT in 8% obese/overweight pts vs. 0% normal body weight pts	assessment of thyroid hormones
Kang et al. [54]	2018	146 pediatric headache pts	39 primary headache pts (16 MIG pts, 18 probable MIG pts, 5 TTH pts); 18 secondary headache pts; 89 unclassified headache pts	mean symptom duration: 5.8 ± 7.9 months, attack frequency: 15.1 ± 10.6 times per month mean pain severity score: 5.1 ± 2.2 in VAS	assessment of thyroid hormones

HT, hypothyroidism; MIG, migraine; Ref., reference; TTH, tension-type headache; VAS, visual analog scale; vs., versus.

## Abbreviations

ACTH	adrenocorticotropic hormone
BMI	body mass index
CGRP	calcitonin gene-related peptide
CM	chronic migraine
EM	episodic migraine
GH	growth hormone
HAH	hypothyroidism-associated headaches
HD	Hashimoto’s disease
ICHD	International Classification of Headache Disorders
MIDAS	Migraine Disability Assessment Score
NDPH	new daily persistent headaches
PRISMA	Preferred Research Items for Systematic Reviews and Meta-Analyses
TRH	thyrotropin-releasing hormone
TSH	thyroid-stimulating hormone
TTH	tension-type headache
